# Measurement of Homocitrulline, A Carbamylation‐derived Product, in Serum and Tissues by LC‐MS/MS

**DOI:** 10.1002/cpz1.762

**Published:** 2023-04-25

**Authors:** Stéphane Jaisson, Aurore Desmons, Manon Doué, Laëtitia Gorisse, Christine Pietrement, Philippe Gillery

**Affiliations:** ^1^ Laboratory of Biochemistry and Molecular Biology, CNRS/URCA UMR N°7369 Extracellular Matrix and Cell Dynamics University of Reims Champagne‐Ardenne Reims France; ^2^ Laboratory of Pediatric Biology and Research University Hospital of Reims Reims France; ^3^ Department of Pediatrics (Nephrology unit) University Hospital of Reims Reims France

**Keywords:** carbamylation, homocitrulline, LC‐MS/MS, protein molecular aging

## Abstract

Carbamylation corresponds to the nonenzymatic binding of isocyanic acid to protein amino groups and participates in protein molecular aging, characterized by the alteration of their structural and functional properties. Carbamylated proteins exert deleterious effects *in vivo* and are involved in the progression of various diseases, including atherosclerosis and chronic kidney disease. Therefore, there is a growing interest in evaluating the carbamylation rate of blood or tissue proteins, since carbamylation‐derived products (CDPs) constitute valuable biomarkers in these contexts. Homocitrulline, formed by isocyanic acid covalently attaching to the ε‐NH_2_ group of lysine residue side chain, is the most characteristic CDP. Sensitive and specific quantification of homocitrulline requires mass spectrometry–based methods. This article describes a liquid chromatography–tandem mass spectrometry (LC‐MS/MS) method for the quantification of homocitrulline, with special emphasis on preanalytical steps that allow quantification of total or protein‐bound homocitrulline in serum or tissue samples. © 2023 The Authors. Current Protocols published by Wiley Periodicals LLC.

**Basic Protocol 1**: Sample pretreatment for the quantification of homocitrulline by LC‐MS/MS

**Alternate Protocol**: Preanalytical steps for the quantification of homocitrulline in tissue samples

**Basic Protocol 2**: LC‐MS/MS quantification of homocitrulline

**Basic Protocol 3**: LC‐MS/MS quantification of lysine in hydrolysates

## INTRODUCTION

Protein molecular aging, which is characterized by progressive and irreversible alterations of protein structure and function, is mainly explained by the accumulation of nonenzymatic post‐translational modifications the proteins are exposed to during their biological life. These modifications correspond to the binding of small metabolites to free reactive groups of amino acid residues, usually followed by subsequent molecular rearrangements (Jaisson & Gillery, 2010). In the case of carbamylation, isocyanic acid reacts in an irreversible manner with α‐ and ε‐amino groups of proteins, generating α‐carbamylated proteins and homocitrulline (HCit, ε‐carbamoyllysine) residues, respectively (Kraus & Kraus, 2001). *In vivo*, isocyanic acid is formed by spontaneous dissociation of urea into cyanate and ammonia (Kraus & Kraus, 2001), by myeloperoxidase‐mediated transformation of thiocyanate (Wang et al., 2007), and to a lesser extent by exogenous sources from biomass burning or tobacco smoke (Roberts et al., 2011).

It is now well established that carbamylated proteins constitute a molecular substratum for many dysfunctions described in chronic diseases, such as chronic kidney disease, atherosclerosis, or rheumatoid arthritis (Kalim et al., 2014; Mastrangelo et al., 2015; Verbrugge et al., 2015). Accordingly, carbamylation‐derived products (CDPs) are considered relevant biomarkers of these diseases. For example, elevated serum concentrations of HCit are associated with mortality and adverse outcomes in end‐stage kidney disease (Koeth et al., 2013) and in various cardiovascular diseases (Tang et al., 2013; Wang et al., 2007).

The quantification of HCit has long been a challenge because most conventional methods were not sensitive enough to detect the limited extent of protein carbamylation. For example, colorimetric assays based on the reactivity of carbamoyl groups with diacetylmonoxime have been described (Balion et al., 1998) but suffered from analytical interference when performed in complex matrices such as plasma. Similar limitations were encountered with immunoassays (Apostolov et al., 2005) because of a lack of standardization and especially the use of home‐made antibodies, which are less specific and poorly characterized. Mass spectrometry–based methods are now considered the gold standard for the quantification of protein molecular aging markers, such as CDPs or advanced glycation end‐products, because they offer excellent specificity and sensitivity.

Herein, we describe a liquid chromatography coupled to tandem mass spectrometry (LC‐MS/MS) method to quantify HCit in serum and tissue samples (Jaisson et al., 2012). This method is divided into three parts. Basic Protocol 1 and Alternate Protocol describe preanalytical steps to quantify HCit in its different forms (total or protein bound) and in different matrices (serum, plasma, or tissue extracts), including details about the acid hydrolysis step, which is compulsory for the cleavage of peptide bonds and the release of HCit residues. Basic Protocol 2 describes the chromatographic separation based on a hydrophilic interaction liquid chromatography (HILIC) and mass detection using a triple quadrupole (e.g., TSQ Quantis, Thermo Scientific). Basic Protocol 3 describes the quantification of lysine (Lys) residues in hydrolysates, since HCit results are generally expressed as a ratio to Lys concentrations (Koeth et al., 2013; Wang et al., 2007).

*NOTE*: All protocols using live animals or animal samples must first be reviewed and approved by an Institutional Animal Care and Use Committee (IACUC) or conform to local guidelines regarding the care and use of laboratory animals.

*NOTE*: All protocols involving humans or human samples must first be reviewed and approved by an Institutional Review Board (IRB) or must follow local guidelines for the use of human samples. All participants must provide informed consent.

## Basic Protocol 1 SAMPLE PRETREATMENT FOR THE QUANTIFICATION OF HOMOCITRULLINE BY LC‐MS/MS

This protocol describes sample pretreatment, including homogenization of tissue samples; dialysis or protein precipitation steps, which allow the quantification of protein‐bound HCit; and acid hydrolysis, which is compulsory for the cleavage of peptide bounds and release of HCit residues.

### Materials

Serum or plasma samples
0.15 M NaCl (87.66 g NaCl in 10 L distilled water; e.g., Euromedex)
20% (w/v) trichloracetic acid (e.g., Sigma‐Aldrich)
6 and 12 M HCl (e.g., Merck)
5.26 mM HCit (1 g/L N^ε^‐carbamoyl‐Lys; e.g., Sigma‐Aldrich)
200 µM d_3_‐l‐HCit (e.g., CDN Isotopes)
125 mM ammonium formate (see recipe)

GeBAflex mini dialysis unit, 8‐kDa cutoff, 10 to 250 µl (e.g., Dominique Dutscher)
12‐L bucket
0.2‐ and 1.5‐ml microcentrifuge tubes
Vortex mixer
Microcentrifuge
2‐ml glass ampoules for cell freezing in liquid nitrogen (e.g., Dominique Dutscher)
Bunsen burner
110°C oven
Evaporator for microcentrifuge tubes connected to a nitrogen stream (e.g., Dominique Dutscher)
Chemical hood
Orbital shaker for microcentrifuge tubes
0.45‐µm polytetrafluoroethylene (PTFE) filters, 4‐mm diameter (e.g., Phenomenex)
Vials for liquid chromatography

*NOTE*: Total HCit in serum or in plasma samples obtained from EDTA‐ or lithium heparinate‒containing tubes can be quantified. Serum and plasma samples should be stored at –80°C. Quantification of protein‐bound HCit requires preanalytical dialysis or protein precipitation to remove free and peptide‐bound HCit. Though it takes longer, we prefer dialysis because precipitated proteins can be difficult to resuspend resulting in lower yields.

#### Dialyze serum/plasma samples to remove free and peptide‐bound HCit

Dialysis removes free HCit and peptide‐bound HCit, yielding samples containing only macromolecules (including proteins), so that only protein‐bound HCit will be quantified.

1a Transfer 150 µl serum or plasma into a GeBAflex mini dialysis unit with 8‐kDa cutoff.
2a Using the foam support provided with the kit, place GeBAflex dialysis unit into a bucket containing 10 L of 0.15 M NaCl.
  Do not exceed 20 samples per bucket. If there are more samples, use additional buckets.
3a Dialyze for 24 hr at 4°C with constant stirring or agitation, and replace dialysate twice during dialysis.
4a Remove dialyzed serum from the dialysis unit, and transfer to a 1.5‐ml microcentrifuge tube.
  Dialyzed samples may be stored at –80°C before acid hydrolysis.

#### Precipitate proteins from samples to remove free and peptide‐bound HCit

**Figure 1:**
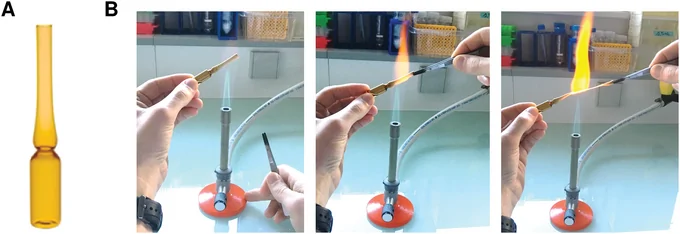
Sealing of 2‐ml glass ampoules before acid hydrolysis. (**A**) 2 ml‐glass ampoules used for acid hydrolysis. (**B**) When sealing ampoules, do not wear gloves (which increases risk of burn). Tilt the ampoule to 45° above the Bunsen burner (focusing on the cone formed by the flame), and wait several seconds until the glass turns orange in color. Then gently pull with pliers.

1b Place 30 µl serum or plasma into a 1.5‐ml microcentrifuge tube. Add 220 µl Milli‐Q‐purified water and 750 µl of 20% (w/v) trichloracetic acid.
2b Shake tubes for 30 s using a vortex mixer. Place tubes on ice for 10 min, and then pellet proteins by centrifugation for 10 min at 10,000 × *g*, 4°C.
3b Gently discard supernatant, and resuspend pellet in 1.2 ml of 6 M HCl by shaking for 10 s in a vortex mixer.
4b Transfer solution to a 2‐ml glass ampoule, and seal glass ampoule using a Bunsen burner (Fig. 1).
  Follow the next steps described in the acid hydrolysis section, beginning at step 12.

#### Prepare calibrators

Calibrators are prepared by spiking a diluted serum (or plasma) sample (matrix blank [MB]) with different amounts of HCit standard. The use of samples made of a similar matrix to patient samples is compulsory to ensure the HCit standard behaves the same as the sample during separation by LC. The HCit concentration contained in the MB will be subtracted from values of the calibration points.

5 Prepare MB solution by diluting 30 µl serum (or plasma) with 2.97 ml of 0.15 M NaCl.
  This serum (or plasma) sample should be randomly selected from available serum (or plasma) samples deriving from control participants. Serum (or plasma) from patients with renal failure should be avoided.
6 Prepare 200 µM HCit (high‐level calibrator) by mixing 5 µl serum (or plasma) used to prepare MB, 18.9 µl of 5.29 mM HCit (1 g/L), and 476.1 µl of 0.15 M NaCl.
7 Prepare a two‐fold dilution series of the calibrator in MB solution to obtain six HCit concentrations: 200, 100, 50, 25, 12.5, and 6.25 µM. To do this, mix 100 µl of 200 µM calibrator with 100 µl MB solution to make 100 µM calibrator, and then combine 100 µl of 100 µM calibrator with 100 µl MB solution to make 50 µM calibrator. Repeat until a 6.25 μM solution is prepared.
8 Store calibrators and MB solution at –80°C until ready for acid hydrolysis.

#### Perform acid hydrolysis and evaporate hydrolysates

9 Add 30 µl serum/plasma sample (or calibrator) to 6 µl of 200 µM d_3_‐HCit (internal standard [IS]) and 564 µl Milli‐Q water into a 1.5‐ml microcentrifuge tube.
  If concentrations of HCit are expected to be very high, use 15 µl serum/plasma instead of 30 µl, and make up the volume by adding 15 µl of 0.15 M NaCl. The results will be multiplied by two during calculation steps.
10 Transfer 0.6 ml solutions into 2‐ml glass ampoules, and add 0.6 ml of 12 M HCl to each ampoule.
11 Seal glass ampoules with a Bunsen burner (Fig. 1).
  CAUTION: Be careful not to burn yourself when sealing the glass ampoules (which remain very hot a few minutes after sealing). Hold the bottom of the ampoule. Do not wear latex or nitrile gloves while using the Bunsen burner, as it will increase the risk of harm.
12 Incubate ampoules for 18 hr in a 110°C oven.
  CAUTION: Comply with the operating instructions of the oven, and be careful not to burn yourself.
13 Cool ampoules on the bench for 30 min. Then break glass ampoules, and transfer 1 ml of each hydrolysate to new 1.5‐ml microcentrifuge tubes.
14 Transfer tubes to an evaporator connected to a nitrogen gas inlet and located under a chemical hood.
15 Evaporate samples until dry (∼8 hr). Add 1 ml Milli‐Q water to each sample, and evaporate overnight to ensure complete removal of HCl.
  After evaporation, the tube walls will look brown.
  The dried samples may be stored at –80°C before the next steps.
16 Add 100 µl of 125 mM ammonium formate to each dried hydrolysate.
17 Shake tubes for 10 s on a vortex mixer. Transfer tubes to an orbital shaker, and agitate for 10 min at 1400 rpm.
18 Centrifuge tubes 5 min at 12,000 × *g*, 20°C.
19 Gently collect 50 µl supernatant, and filter through a 0.45‐µm PTFE syringe filter into a 0.2‐ml microcentrifuge tube. Repeat with the remaining 50 µl supernatant.
  Filtration should be performed in two steps (two filtrations of 50 µl). Transfer 50 µl into the filter, and then attach a 1‐ml syringe to the filter. Push the plunger to force the liquid through the filter. Use a new filter for each sample, and wash the syringe with distilled water between each sample (taking care to dry the syringe with a paper towel to avoid any dilution of the sample).
20 Dilute filtered hydrolysate 10‐fold with 125 mM ammonium formate (10 µl hydrolysate + 90 µl of 125 mM ammonium formate), and transfer to an appropriate chromatography vial.
  The diluted hydrolysates may be stored at –80°C until ready for LC‐MS/MS analysis.

## PREANALYTICAL STEPS FOR THE QUANTIFICATION OF HOMOCITRULLINE IN TISSUE SAMPLES

Quantification of HCit may be performed in all types of tissues (fresh or frozen), including bones. In the case of frozen tissues, the samples should be cut into small pieces (100 to 150 mg each) and stored at –80°C until analysis. Using this protocol, only total HCit can be quantified.

### Additional Materials (also see Basic Protocol 1)

Fresh or frozen tissue samples
0.5 M acetic acid (e.g., Sigma‐Aldrich)
200 IU pepsin, specific activity >3000 IU/mg (e.g., Sigma‐Aldrich)

Lysing Matrix tubes, A and D (e.g., MP Biomedicals)
FastPrep‐24 (e.g., MP Biomedicals)
37°C incubator

#### Homogenize tissue samples

1 Place 100 to 150 mg fresh or frozen tissue samples into Lysing Matrix tubes, and add 1 ml of 0.5 M acetic acid to each sample.
  The choice of Lysing Matrix tube depends on the stiffness of the tissue. For example, use Lysing Matrix D for skin, vessels, kidney, or liver. Use Lysing Matrix A for bone. For more details, refer to MP Biomedicals website (https://www.mpbio.com).
2 Transfer tubes into the FastPrep‐24 system.
3 Perform four cycles of 40 s at 6 m/s. Between each cycle, place tubes on ice for 5 min.
4 Centrifuge Lysing Matrix tubes 5 min at 14,000 × *g*, 4°C.
5 Gently collect supernatant (about 800 µl). Add 200 IU pepsin, and incubate for 24 hr at 37°C.
  Predigestion of proteins with pepsin improves the efficiency of acid hydrolysis.
6 Centrifuge tubes 5 min at 10,000 × *g*, 4°C.
7 Collect supernatant (about 800 µl), and dilute two‐fold with Milli‐Q water (i.e., add 800 µl).
  Store the diluted homogenate at –80°C until ready for acid hydrolysis.

#### Prepare calibrators

8 Prepare calibrators as described in Basic Protocol 1, steps 5 to 8.
  In this protocol, the IS d_3_‐HCit is added after acid hydrolysis.
  Store each calibrator and MB solution at –80°C until ready for acid hydrolysis.

#### Perform acid hydrolysis and evaporae hydrolysates

9 Transfer 0.6 ml diluted tissue homogenates into 2‐ml glass ampoules, and add 0.6 ml of 12 M HCl to each ampoule.
10 Seal glass ampoules with a Bunsen burner (Fig. 1).
  CAUTION: Be careful not to burn yourself when sealing the glass ampoules (which remain very hot a few minutes after sealing). Hold the bottom of the ampoule, and do not wear latex or nitrile gloves while using the Bunsen burner.
11 Incubate ampoules for 18 hr in a 110°C oven.
  CAUTION: Comply with the operating instructions of the oven, and be careful not to burn yourself.
12 Cool ampoules on the bench for 30 min.
  After hydrolysis of the tissue samples, some black sediment may appear in the ampoule. These sediments correspond to tissue fragments that have not been hydrolyzed because there is too much material in the ampoule. If there is excessive sediment, the experiment must be repeated using a higher dilution of sample prior to hydrolysis in order to place less material in the ampoule.
13 Break glass ampoules, and transfer 1 ml of each hydrolysate to new 1.5‐ml microcentrifuge tubes.
14 Transfer microcentrifuge tubes to an evaporator connected to a nitrogen gas inlet and located under a chemical hood.
15 Evaporate samples until dry (∼8 hr). Add 1 ml Milli‐Q water to each sample, and evaporate overnight to ensure complete removal of HCl.
  After evaporation, the tube walls will look brown.
  Dried samples may be stored at –80°C until ready to perform the next steps.
16 Add 100 µl of 125 mM ammonium formate containing 1 µM d_3_‐HCit to each dried hydrolysate.
17 Shake tubes for 10 s on a vortex mixer. Transfer tubes to an orbital shaker, and agitate for 10 min at 1400 rpm.
18 Centrifuge tubes 5 min at 12,000 × *g*, 20°C.
19 Gently collect 50 µl supernatant, and filter through a 0.45‐µm PTFE syringe filter into a 0.2‐ml microcentrifuge tube. Repeat with the remaining 50 µl supernatant.
  Filtration should be performed in two steps (two filtrations of 50 µl). Transfer 50 µl into the filter. Then attach a 1‐ml syringe to the filter, and push the plunger to force the liquid through the filter. Use a new filter for each sample, and wash the syringe with distilled water between each sample (taking care to dry the syringe with a paper towel to avoid any dilution of the sample). However, it is possible to use a new syringe for each sample.
20a Dilute filtered sample hydrolysates with 125 mM ammonium formate containing 1 µM d_3_‐HCit, and transfer to an appropriate chromatography vial.
  Preliminary tests should be performed to determine the best dilution factor, which depends on the HCit content in the tissue and on the sensitivity of the mass spectrometer. Usual dilution rates range from 1:5 to 1:20 (v/v).
20b Dilute filtered calibrator hydrolysates 10‐fold with 125 mM ammonium formate containing 1 µM d_3_‐HCit.
  After the 10‐fold dilution of calibrator hydrolysates, the final HCit concentrations of calibrators will be: 5, 2.5, 1.25, 0.625, 0.312, and 0.156 µM.
  Diluted hydrolysates may be stored at –80°C until LC‐MS/MS analysis.

## Basic Protocol 2 LC‐MS/MS QUANTIFICATION OF HOMOCITRULLINE

This protocol describes LC‐MS/MS analysis of protein hydrolysates to quantify HCit.

### Materials

5 mM ammonium formate, pH 2.9 (see recipe)
Acetonitrile (e.g., VWR)
Formic acid (e.g., Merck)

Vials for liquid chromatography (e.g., VWR)
LC system (e.g., Ultimate 3000, Thermo Scientific)
Triple quadrupole detector/electrospray ion source (e.g., TSQ Quantis, Thermo Scientific)
100 × 4.6‐mm Kinetex HILIC column, packed with 2.6‐µm particles (e.g., Phenomenex)
Syringe pump (e.g., Happaratus)
Tracefinder v. 4.1 (e.g., Thermo Scientific)

LC‐MS/MS quantification of HCit is performed using a triple quadrupole MS detector. We use a TSQ Quantis triple quadrupole tandem mass spectrometer interfaced with an electrospray ionisation source. Separation is performed using a 50 × 2.1–mm Acquity UPLC BEH HILIC column (unbound silica with reticulated diol groups on its surface), packed with 1.7‐µm particles preceded by a prefilter. Mobile phase A is a 5 mM ammonium formate (pH 2.9), and mobile phase B is 100% (v/v) acetonitrile + 0.1% formic acid. Details about the buffer gradient, flow rate, and injection volume are provided in Table 1.

**Table 1:** Liquid Chromatography Parameters for the Separation of HCit Using the Acquity UPLC BEH HILIC Column

Parameter	Specification
Flow rate	0.3 ml/min
Mobile phase A	5 mM ammonium formate, pH 2.9
Mobile phase B	100% acetonitrile + 0.1% formic acid
Autosampler temperature	8°C
Injection volume	3 µl
% B for column equilibration^*a*^	90%
Gradient	0 → 0.75 min, 90% B
	0.75 → 1.25 min, linear gradient to 5% B
	1.25 → 2.25 min, 5% B
	2.25 → 3.0 min, linear gradient to 90% B
	3.0 → 4.0 min, 90% B
Oven temperature	30°C
Rotary valve^*b*^	0 → 0.5 min, position 1 (→ waste)
	0.5 → 3.5 min, position 2 (→ MS)
	3.5 → 4.0 min, position 1 (→ waste)

HCit, homocitrulline; HILIC, hydrophilic interaction liquid chromatography; MS, mass spectrometry; UPLC, ultra‐performance liquid chromatography.

^*a*^ Equilibration time with 90% B, 30 min.

^*b*^ The rotary valve is located in the oven, just after the column. The valve is not essential, but it helps prevent the mass spectrometer ion source from clogging and ensures robust results.

Ion source and mass detection parameters are detailed in Tables 2 and 3, respectively. The multiple reaction monitoring (MRM) transition 190.2 → 127.1 is used as the quantification transition, whereas 190.2 → 173.1 is used to confirm peak specificity. MRM transition 193.2 → 176.1 is used for IS (i.e., d_3_‐HCit) quantification. Note that these mass spectrometry parameters have been optimized for this specific system and should be redefined if using a different mass spectrometer. For that purpose, directly infuse the standard solution of each analyte (HCit and IS) into the mass spectrometer source using a syringe pump.

**Table 2:** Ion Source and Gas Parameters for the Detection of HCit Using the TSQ Quantis MS/MS System

Ionization type	Electrospray (H‐ESI)
Mode	Positive
Source position	M/1/central
Spray voltage	Static
Positive ion	4000 V
Negative ion	2500 V
Sheath gas	25 a.u.
Aux gas	2 a.u.
Sweep gas	0 a.u.
Ion transfer tube temperature	250°C
Vaporizer temperature	300°C

a.u., arbitrary units; HCit, homocitrulline; H‐ESI, heated electrospray ionization; MS/MS, tandem mass spectrometry.

Nitrogen is used as curtain and nebulization gas, and argon is used as collision gas.

**Table 3:** Mass Spectrometry Parameters for the MRM Detection of HCit Using the TSQ Quantis MS/MS System

Compound	Precursor ion (*m/z*)	Product ion (*m/z*)	Collision energy (V)	Dwell time (ms)
HCit	190.2	127.1	16	200
		173.1	11	200
d_3_‐HCit (IS)	193.2	176.1	11	200

CID, collision‐induced dissociation; FWHM, full width at half maximum; HCit, homocitrulline; IS, internal standard; MRM, multiple reaction monitoring; MS/MS, tandem mass spectrometry.

Other parameters: Q1 resolution (FWHM), 0.7; Q3 resolution (FWHM), 1.2; CID gas (mTorr), 1.5; source fragmentation, 0; chromatographic peak width, 6.

**Figure 2:**
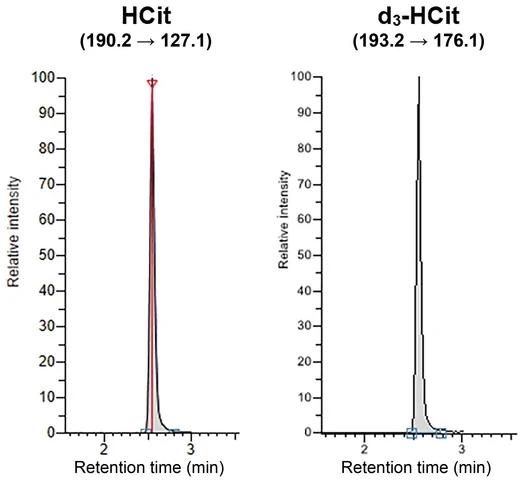
Typical LC‐MS/MS chromatogram obtained for homocitrulline (HCit) assay using a serum sample

Before each series, inject two blank samples (5 mM ammonium formate, pH 2.9) to condition the column. Insert a blank sample after the highest concentration calibrator and after samples with very high HCit concentration to avoid any cross‐over contamination. An example of a chromatogram obtained by analyzing a serum sample is shown in Figure 2. HCit and its IS coelute at a retention time of 2.97 min.

**Figure 3:**
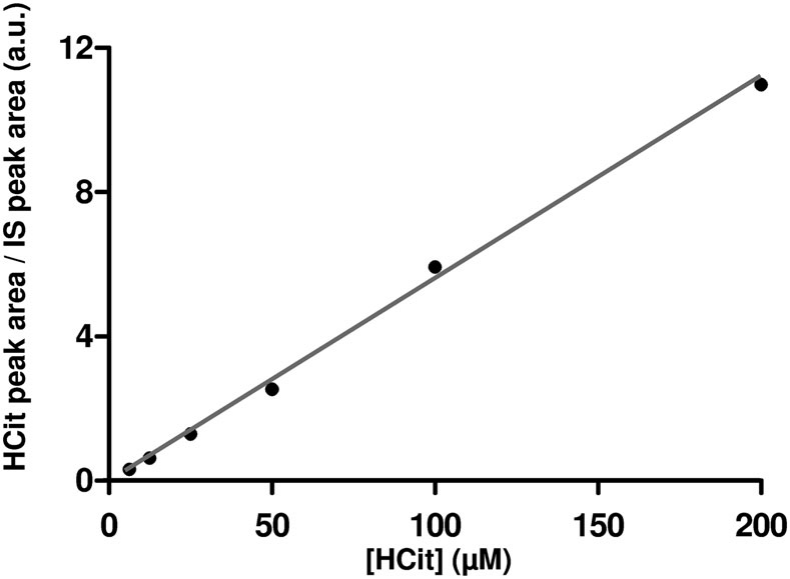
Example calibration curve used for the quantification of serum HCit. Regression equation: *y* = 0.0561 × –0.0316 (*r*2 = 0.995). a.u., arbitrary units; HCit, homocitrulline; IS, internal standard.

Quantification of peak area is performed using Tracefinder 4.1 software. Ratios of HCit quantification peak area to IS peak area are used to derive the calibration curve and determine HCit concentrations in the samples. An example calibration curve is shown in Figure 3. When establishing the calibration curve, the HCit‐to‐IS ratio of the matrix blank sample must be subtracted from the ratios of all the calibrators before calculation.

To ensure specificity of quantification, the ratio of areas of HCit quantification peak (190.2 → 127.1) to HCit confirmation peak (190.2 → 173.1) must be between 0.6 and 0.8. Each sample should be injected twice, and mean peak areas should be used for calculations.

## Basic Protocol 3 LC‐MS/MS QUANTIFICATION OF LYSINE IN HYDROLYSATES

In order to evaluate the rate of protein carbamylation, it is important to normalize results toward the protein content in the serum sample or in the tissue extract. Thus, it is possible to perform usual protein quantification assays and to report HCit results expressed as mmol/g protein. However, most studies dealing with protein carbamylation express HCit results as a ratio to Lys content. Accordingly, here we describe an LC‐MS/MS method for the quantification of Lys in hydrolysates.

### Additional Materials (also see Basic Protocols 1 and 2)

Filtered hydrolysates (see Basic Protocol 1, step 19)
d_8_‐l‐Lys (CDN isotopes)
d,l‐Lys (Sigma Aldrich)

#### Dilute hydrolysates

For serum and tissue samples, use the filtered hydrolysates obtained after evaporation (i.e., hydrolysates before dilution from Basic Protocol 1, step 19).

1 Dilute filtered hydrolysates 10‐fold with 125 mM ammonium formate containing 65 µM d_8_‐Lys (IS). For example, combine 5 µl hydrolysate with 45 µl buffer.
2 Perform a second dilution using 5 mM ammonium formate (pH 2.9) containing 65 µM d_8_‐Lys.
  Serum samples: Dilute 1:30 (v/v) giving a final dilution of 1:300.
  Tissue samples: Dilute 1:10 to 1:100 (v/v) giving a final dilution between 1:100 and 1:1000.
  The second dilution may be done directly in chromatography vials or in 96‐well plates; both vials and plates may be used for injection.
  The second dilution is performed with 5 mM ammonium formate with a pH at 2.9 to keep the pH close to the pH of mobile phase A.
  The dilution factor for tissue will depend on the type of tissue and should be determined empirically.
  The diluted hydrolysates may be stored at –80°C until LC‐MS/MS analysis.

#### Prepare calibrators for lysine quantification

The calibration curve for Lys quantification is calculated from calibrators prepared by spiking Lys standard into an MB sample prepared using diluted serum (or plasma) hydrolysate. However, it is not compulsory to submit calibrators to acid hydrolysis for establishing Lys calibration curve.

3 Prepare a serum (or plasma) hydrolysate intended to be used as an MB solution. For that purpose, perform Basic Protocol 1, steps 9 to 19 (i.e., acid hydrolysis of a serum [or plasma] sample [without adding IS] until hydrolysate filtration).
  This serum (or plasma) sample can be selected randomly among the samples available in the laboratory.
4 Dilute 50 µl MB serum hydrolysate with 0.95 ml of 125 mM ammonium formate.
5 Prepare 30 mM Lys (high‐level calibrator) by mixing 10 µl MB serum hydrolysate, 60 µl of 0.1 M (14.6 mg/ml) Lys solution, and 130 µl of 125 mM ammonium formate.
6 Prepare a two‐fold dilution series of the calibrator in MB solution to obtain five Lys concentrations: 30, 15, 7.5, 3.75, and 1.875 mM. To do this, mix 100 µl of 30 mM calibrator with 100 µl MB solution to make 15 mM calibrator, and then combine 100 µl of 15 mM calibrator with 100 µl MB solution. Repeat until a 1.875 mM solution is prepared.
7 Dilute each calibrator 10‐fold with 125 mM ammonium formate (pH 2.9) containing 65 µM d_8_‐Lys. For example, dilute 5 µl hydrolysate in 45 µl buffer.
8 Dilute each calibrator 30‐fold with 5 mM ammonium formate (pH 2.9) containing 65 µM d_8_‐Lys to obtain final concentrations of 100, 50, 25, 12.5, and 6.25 µM Lys.
  The calibrators may be stored at –80°C until LC‐MS/MS analysis.

#### Quantify Lys by LC‐MS/MS

The same LC‐MS/MS system, LC column, and mobile phases as for HCit quantification (Basic Protocol 2) are used for the quantification of Lys. Details about the buffer gradient, flow rate, and injection volume are shown in Table 4.

**Table 4:** Liquid Chromatography Parameters for the Separation of Lys Using the Acquity UPLC BEH HILIC Column

Parameter	Specification
Flow rate	0.2 ml/min
Mobile phase A	5 mM ammonium formate, pH 2.9
Mobile phase B	100% acetonitrile + 0.1% formic acid
Autosampler temperature	8°C
Injection volume	1 µl
%B for column equilibration^*a*^	80%
Gradient	0 → 1.0 min, 80% B, flow rate 0.2 ml/min
	1.0 → 1.125 min, linear gradient to 30% B, flow rate 0.4 ml/min
	1.125 → 2.25 min, linear gradient to 5% B, flow rate 0.4 ml/min
	2.25 → 2.75 min, 5% B, flow rate 0.4 ml/min
	2.75 → 3.25 min, linear gradient to 80% B, flow rate 0.2 ml/min
	3.25 → 4.0 min, 80% B, flow rate 0.2 ml/min
Oven temperature	30°C

HILIC, hydrophilic interaction liquid chromatography; Lys, lysine; MS, mass spectrometry; UPLC, ultra‐performance liquid chromatography.

^*a*^ Equilibration time with 80% B, 30 min.

Ion source and mass detection parameters are detailed in Tables 5 and 6, respectively. The MRM transition 147.1 → 84.1 is used as the quantification transition, whereas 147.1 → 130.1 is used to confirm peak specificity. MRM transition 155.2 → 92.2 is used for IS (i.e., d_8_‐Lys) quantification. As with HCit quantification, these parameters have been optimized for this mass spectrometry system and should be redefined if another mass spectrometer is used. For that purpose, directly infuse the standard solution of each analyte (Lys and IS) into the mass spectrometer source using a syringe pump.

**Table 5:** Ion Source and Gas Parameters for the Detection of Lys Using the TSQ Quantis MS/MS System

Ionization type	Electrospray (H‐ESI)
Mode	Positive
Source position	M/1/central
Spray voltage	Static
Positive ion	4000 V
Negative ion	2500 V
Sheath gas	40 a.u.
Aux gas	5 a.u.
Sweep gas	0 a.u.
Ion transfer tube temperature	300°C
Vaporizer temperature	250°C

a.u., arbitrary units; H‐ESI, heated electrospray ionization; Lys, lysine; MS/MS, tandem mass spectrometry.

Nitrogen is used as curtain and nebulization gas, and argon is used as collision gas

**Table 6:** Mass Spectrometry Parameters for the MRM Detection of Lys Using the TSQ Quantis MS/MS System

Compound	Precursor ion (*m/z*)	Product ion (*m/z*)	Collision energy (V)	Dwell time (ms)
Lys	147.1	84.1	16	450
		130.1	10	450
d_8_‐Lys (IS)	155.2	92.2	19	450

CID, collision‐induced dissociation; FWHM, full width at half maximum; IS, internal standard; Lys, lysine; MRM, multiple reaction monitoring; MS/MS, tandem mass spectrometry.

Other parameters: Q1 resolution (FWHM), 0.7; Q3 resolution (FWHM), 1.2; CID gas (mTorr), 1.5; source fragmentation, 0; chromatographic peak width, 6.

Before each series, inject two blank samples (5 mM ammonium formate, pH 2.9) to condition the column. Insert a blank sample after the calibrator with the highest concentration and after samples with very high Lys concentration to avoid any cross‐over contamination.

Quantification of peak area is performed using Tracefinder 4.1 software. Ratios of Lys quantification peak area to IS peak area are used to calculate the calibration curve and to determine Lys concentrations in the samples. When establishing the calibration curve, the Lys‐to‐IS ratio of the matrix blank sample has to be subtracted from the ratios of all the calibrators before calculation.

To ensure specificity of quantification, the ratio of areas of Lys quantification peak (147.1 → 84.1) to Lys confirmation peak (147.1 → 130.1) must be between 2.1 and 2.3. Each sample should be injected twice, and mean peak areas should be used for calculations. For the calculation of Lys concentrations used for the expression of HCit results, be sure to use the correct dilution factor. For serum (or plasma) samples, the dilution factor is 1:1200 (v/v): 1:40 dilution through acid hydrolysis, 10‐fold concentration after evaporation (from 1 ml to 100 µl), and 1:300 dilution before LC‐M/MS. For tissue samples, it is easier to use HCit results corresponding to HCit concentrations in the undiluted hydrolysates. In this case, dilution factors for Lys concentrations range from 100 to 1000.

## REAGENTS AND SOLUTIONS

### Ammonium formate buffer, 5 mM (pH 2.9)

20 ml of 125 mM ammonium formate (e.g., Sigma‐Aldrich)
450 ml Milli‐Q water
Adjust pH to 2.9 with formic acid
Bring volume to 500 ml with Milli‐Q water
Store at 4°C for up to 15 days

### Ammonium formate buffer, 5 mM (pH 2.9), containing 65 µM d_8_‐Lys

10 µl of 650 mM (100 mg/ml) d_8_‐Lys
100 ml of 5 mM ammonium formate, pH 2.9 (see recipe)
Store at –20°C for up to 6 months
  The solution can be frozen and thawed several times.

### Ammonium formate, 125 mM

788.3 mg ammonium formate
100 ml water
Do not adjust pH
Store at –20°C for up to 6 months
  The solution can be frozen and thawed several times.

### Ammonium formate, 125 mM, containing 1 µM d_3_‐HCit

788.3 mg ammonium formate
200 µl of 500 µM d_3_‐HCit solution, obtained from 1:100 (v/v) dilution of 50 mM (9.1 g/L) d_3_‐HCit
9.8 ml water
Do not adjust pH
Store at –20°C for up to 6 months
  The solution can be frozen and thawed several times.

### Ammonium formate, 125 mM, containing 65 µM d_8_‐Lys

788.3 mg ammonium formate
10 µl of 650 mM (100 mg/ml) d_8_‐Lys
9.8 ml water
Do not adjust pH
Store at –20°C for up to 6 months
  The solution can be frozen and thawed several times.

## COMMENTARY

### Background Information

HCit is a general marker of protein carbamylation formed by the binding of isocyanic acid to ε‐NH2 group of Lys side chains. As carbamylated proteins have been clearly identified as contributing factors in the development of long‐term complications related to chronic diseases (Verbrugge et al., 2015), CDPs (e.g., HCit) are emerging biomarkers. Various studies have pointed out the added value of HCit quantification in different pathological contexts, including chronic kidney disease or cardiovascular diseases (Jaisson et al., 2015; Koeth et al., 2013).

Accordingly, it is essential to use robust analytical methods for the quantification of carbamylation biomarkers to boost their use in clinical studies. Few methods have been described so far, and most rely on colorimetry, immunoassays, or mass spectrometry. Colorimetric techniques lack the sensitivity and specificity needed to quantify carbamyltion in complex matrices such as serum. Immunoassays may constitute alternate methods but suffer from a lack of standardization (i.e., a lack of commercially available HCit antibodies). Indeed, the only immunoassays described use home‐made antibodies, which are poorly characterized. As a consequence, LC‐MS/MS methods have emerged as the gold standard for the quantification of CDPs, especially owing to their high sensitivity and specificity.

The disadvantage of LC‐MS methods over colorimetric or immunological assays is that they only enable the quantification of HCit in its free amino acid form. Since it is necessary to quantify protein‐bound HCit (and not free HCit) for evaluating protein carbamylation rate, HCit residues must be released from proteins by cleaving peptide bonds. This requires important preanalytical steps, descriptions of which are often only summarized in publications. Thus, the goal of this protocol is to detail these different steps.

The first step of the experimental design is to determine which HCit form has to be assayed (total or protein bound). The quantification of protein‐bound HCit requires the removal of free HCit, either by sample dialysis or by protein precipitation. Both are described in this article. Though more time consuming, we prefer dialysis over protein precipitation. Indeed, the protein pellets obtained by protein precipitation are often difficult to suspend in 6 M HCl, and undissolved portions of the pellet stick to the walls of the tube and are often lost during transfer to the glass ampoules for hydrolysis.

The crucial preanalytical step is acid hydrolysis, which allows the cleavage of peptide bonds. The experimental protocol must be strictly controlled to ensure that all conditions (temperature, incubation time, HCl concentration) are identical for all samples. Indeed, HCit is partially degraded during this step (∼30%), but this degradation rate is constant under given experimental conditions (Jaisson et al., 2012). This problem can therefore be bypassed by a strict adhesion to the protocol. It is also compulsory to subject the calibrators to acid hydrolysis under the same conditions as the assayed samples. An alternative to acid hydrolysis is enzymatic hydrolysis. However, this step is more time consuming, and its main disadvantage is the difficulty of ensuring exhaustive peptide bond cleavage, especially because modified amino acids may limit the action of proteolytic enzymes.

As a conclusion, the LC‐MS/MS method described herein includes many time‐consuming preanalytical steps that are crucial for robust and reliable HCit quantification. Therefore, particular attention must be paid to these steps.

### Critical Parameters and Troubleshooting

*Assay calibration (1)*: As HCit is partially degraded during acid hydrolysis (about 30%), calibrators must be subjected to acid hydrolysis under the same conditions as the samples to avoid underestimation of values. The HCit degradation rate is constant in given experimental conditions, as demonstrated by linearity of the calibration curve (Fig. 3). Moreover, excellent recovery rates are obtained when hydrolyzed calibrators are used for the quantification of HCit (Jaisson et al., 2012).

*Assay calibration (2)*: Calibrators should be prepared by spiking HCit in a diluted serum (or plasma) sample because the presence of matrix is very important for LC separation of HCit. HCit in buffer does not behave like samples (different retention time and peak shape). However, the initial HCit content in the matrix blank must be determined and subtracted from obtained values when calculating the calibration curve.

*Acid hydrolysis*: Take care to strictly respect the incubation time (18 hr) at 110°C. Due to HCit degradation during acid hydrolysis, any variation will affect reliability of the results.

*Evaporation*: It is very important to perform evaporation under a nitrogen stream (to avoid oxidation) until dryness and to perform two successive evaporations (i.e., evaporate HCl, add 1 ml water, and evaporate again) to remove all residual acid. Water must be added only when the samples are totally dry. If evaporation is incomplete or if the acid is not completely removed, the pH of the suspended hydrolysates will be too low and will affect chromatographic separation of HCit (i.e., retention time and peak shape).

*Filtration*: Hydrolysates must be filtered before injection of the sample to avoid clogging the LC system. Use a new filter for each sample, and be careful to correctly wash the syringe and to dry it between each sample to avoid diluting the next hydrolysate.

*Homogenization of tissue samples*: The homogenization time and the type of Lysing Matrix tubes to use may vary depending on the tissue. Preliminary tests should be used to determine the best conditions that will result in homogeneous tissue extracts.

*LC‐MS/MS analysis*: In case of abnormal HCit peaks (asymmetric peak, modification of retention time), check the pH of mobile phase A and the samples (which should range from 4 to 5). If sensitivity is lost, check the ionization source, and clean it if necessary.

*Calculations*: Be very careful to account for the dilution of the hydrolysates and to use the correct concentrations of calibrators (which are different for serum/plasma and tissue samples).

### Understanding Results

Serum HCit concentrations typically range from 100 to 150 µmol/mol Lys in control participants and may reach 1500 µmol/mol Lys in patients with chronic kidney disease (Desmons et al., 2016).

Tissue HCit concentrations may vary depending on the type of tissue. For instance, in mice HCit concentrations range from 200 to 500 µmol/mol Lys in bone, liver, kidney, aorta, and skin. In human skin extracts, HCit concentrations may reach 10,000 µmol/mol Lys in older adults (Gorisse et al., 2016; Pietrement et al., 2013).

### Time Considerations

Dialysis of serum samples takes 1 day. Precipitation of protein (serum/plasma samples) takes 2 hr depending on the number of samples. Tissue homogenization requires 2 days, and acid hydrolysis (including preparation of tubes, sealing of glass ampoules, and incubation) takes 1 day. The evaporation steps require 1 day, and filtration of hydrolysates takes 3 hr depending on the number of samples. LC‐MS/MS analysis takes 2 days, which includes 1 day for HCit quantification and 1 day for Lys quantification. Note that each step may be performed separately, except evaporation, which must be done immediately after acid hydrolysis.

### Author Contributions

**Stéphane Jaisson**: conceptualization, methodology, writing–original draft, writing‒review and editing; **Aurore Desmons**: methodology, writing–review and editing; **Manon Doue**: methodology, writing‒review and editing; **Laëtitia Gorisse**: methodology, writing–review and editing; **Christine Pietrement**: conceptualization, methodology, writing–review and editing; **Philippe Gillery**: conceptualization, supervision, writing–original draft, writing‒review and editing.

### Conflict of Interest

The authors declare no conflicts of interest.

## Data Availability

The data that support the findings of this article are available upon reasonable request from the corresponding author. The data are not publicly available due to privacy or ethical restrictions.

## References

[cpz1762-bib-0001] Apostolov, E. O. , Shah, S. V. , Ok, E. , & Basnakian, A. G. (2005). Quantification of carbamylated LDL in human sera by a new sandwich ELISA. Clinical Chemistry, 51(4), 719–728. 10.1373/clinchem.2004.044032 15684275

[cpz1762-bib-0002] Balion, C. M. , Draisey, T. F. , & Thibert, R. J. (1998). Carbamylated hemoglobin and carbamylated plasma protein in hemodialyzed patients. Kidney International, 53(2), 488–495. 10.1046/j.1523-1755.1998.00777.x 9461111

[cpz1762-bib-0003] Desmons, A. , Jaisson, S. , Pietrement, C. , Rieu, P. , Wynckel, A. , & Gillery, P. (2016). Homocitrulline: A new marker for differentiating acute from chronic renal failure. Clinical Chemistry and Laboratory Medicine, 54(1), 73–79. 10.1515/cclm-2015-0398 26124058

[cpz1762-bib-0004] Gorisse, L. , Pietrement, C. , Vuiblet, V. , Schmelzer, C. E. , Kohler, M. , Duca, L. , Debelle, L. , Fornès, P. , Jaisson, S. , & Gillery, P. (2016). Protein carbamylation is a hallmark of aging. Proceedings of the National Academy of Sciences of the United States of America, 113(5), 1191–1196. 10.1073/pnas.1517096113 26712018 PMC4747743

[cpz1762-bib-0005] Jaisson, S. , & Gillery, P. (2010). Evaluation of nonenzymatic posttranslational modificationderived products as biomarkers of molecular aging of proteins. Clinical Chemistry, 56(9), 1401–1412. 10.1373/clinchem.2010.145201 20562349

[cpz1762-bib-0006] Jaisson, S. , Gorisse, L. , Pietrement, C. , & Gillery, P. (2012). Quantification of plasma homocitrulline using hydrophilic interaction liquid chromatography (HILIC) coupled to tandem mass spectrometry. Analytical and Bioanalytical Chemistry, 402(4), 1635–1641. 10.1007/s00216-011-5619-6 22160237

[cpz1762-bib-0007] Jaisson, S. , Kerkeni, M. , Santos‐Weiss, I. C. , Addad, F. , Hammami, M. , & Gillery, P. (2015). Increased serum homocitrulline concentrations are associated with the severity of coronary artery disease. Clinical Chemistry and Laboratory Medicine, 53(1), 103–110. 10.1515/cclm-2014-0642 25153409

[cpz1762-bib-0008] Kalim, S. , Karumanchi, S. A. , Thadhani, R. I. , & Berg, A. H. (2014). Protein carbamylation in kidney disease: Pathogenesis and clinical implications. American Journal of Kidney Diseases, 64(5), 793–803. 10.1053/j.ajkd.2014.04.034 25037561 PMC4209336

[cpz1762-bib-0009] Koeth, R. A. , Kalantar‐Zadeh, K. , Wang, Z. , Fu, X. , Tang, W. H. , & Hazen, S. L. (2013). Protein carbamylation predicts mortality in ESRD. Journal of the American Society of Nephrology, 24(5), 853–861. 10.1681/ASN.2012030254 23431074 PMC3636787

[cpz1762-bib-0010] Kraus, L. M. , & Kraus, A. P. Jr. (2001). Carbamoylation of amino acids and proteins in uremia. Kidney International. Supplement, 78, S102–107. 10.1046/j.1523-1755.2001.59780102.x 11168993

[cpz1762-bib-0011] Mastrangelo, A. , Colasanti, T. , Barbati, C. , Pecani, A. , Sabatinelli, D. , Pendolino, M. , Truglia, S. , Massaro, L. , Mancini, R. , Miranda, F. , Spinelli, F. R. , Conti, F. , & Alessandri, C. (2015). The role of posttranslational protein modifications in rheumatological diseases: Focus on rheumatoid arthritis. Journal of Immunology Research, 2015, 712490. 10.1155/2015/712490 26090496 PMC4451265

[cpz1762-bib-0012] Pietrement, C. , Gorisse, L. , Jaisson, S. , & Gillery, P. (2013). Chronic increase of urea leads to carbamylated proteins accumulation in tissues in a mouse model of CKD. PLoS One, 8(12), e82506. 10.1371/journal.pone.0082506 24324801 PMC3853192

[cpz1762-bib-0013] Roberts, J. M. , Veres, P. R. , Cochran, A. K. , Warneke, C. , Burling, I. R. , Yokelson, R. J. , Lerner, B. , Gilman, J. B. , Kuster, W. C. , Fall, R. , & de Gouw, J. (2011). Isocyanic acid in the atmosphere and its possible link to smokerelated health effects. Proceedings of the National Academy of Sciences of the United States of America, 108(22), 8966–8971. 10.1073/pnas.1103352108 21576489 PMC3107289

[cpz1762-bib-0014] Tang, W. H. , Shrestha, K. , Wang, Z. , Borowski, A. G. , Troughton, R. W. , Klein, A. L. , & Hazen, S. L. (2013). Protein carbamylation in chronic systolic heart failure: Relationship with renal impairment and adverse long‐term outcomes. Journal of Cardiac Failure, 19(4), 219–224. 10.1016/j.cardfail.2013.02.001 23582087 PMC3635500

[cpz1762-bib-0015] Verbrugge, F. H. , Tang, W. H. , & Hazen, S. L. (2015). Protein carbamylation and cardiovascular disease. Kidney International, 88(3), 474–478. 10.1038/ki.2015.166 26061545 PMC4556561

[cpz1762-bib-0016] Wang, Z. , Nicholls, S. J. , Rodriguez, E. R. , Kummu, O. , Horkko, S. , Barnard, J. , Reynolds, W. F. , Topol, E. J. , DiDonato, J. A. , & Hazen, S. L. (2007). Protein carbamylation links inflammation, smoking, uremia and atherogenesis. Nature Medicine, 13(10), 1176–1184. 10.1038/nm1637 17828273

[cpz1762-bib-0017] Jaisson et al. (2012). See above.

